# Spatiotemporal evolution of COVID-19 in Portugal’s Mainland with self-organizing maps

**DOI:** 10.1186/s12942-022-00322-3

**Published:** 2023-01-29

**Authors:** Igor Duarte, Manuel C. Ribeiro, Maria João Pereira, Pedro Pinto Leite, André Peralta-Santos, Leonardo Azevedo

**Affiliations:** 1 Formely: Instituto Superior Técnico, Universidade de Lisboa, Av. Rovisco Pais, 1049-001 Lisbon, Portugal; 2 Direção de Serviços de Informação e Análise, Direção-Geral da Saúde, Lisbon, Portugal; 3 NOVA National School of Public Health, Public Health Research Centre, Universidade NOVA de Lisboa, Lisbon, Portugal; 4 Comprehensive Health Research Centre (CHRC), Universidade NOVA de Lisboa, Lisbon, Portugal; 5 CERENA/DER, Instituto Superior Técnico, Universidade de Lisboa, Av. Rovisco Pais, 1049-001 Lisbon, Portugal

**Keywords:** Self-organizing maps, COVID-19, Geo-Spatial Analysis, Socio-economic determinants of disease, SARS-CoV-2

## Abstract

**Background:**

Self-Organizing Maps (SOM) are an unsupervised learning clustering and dimensionality reduction algorithm capable of mapping an initial complex high-dimensional data set into a low-dimensional domain, such as a two-dimensional grid of neurons. In the reduced space, the original complex patterns and their interactions can be better visualized, interpreted and understood.

**Methods:**

We use SOM to simultaneously couple the spatial and temporal domains of the COVID-19 evolution in the 278 municipalities of mainland Portugal during the first year of the pandemic. Temporal 14-days cumulative incidence time series along with socio-economic and demographic indicators per municipality were analyzed with SOM to identify regions of the country with similar behavior and infer the possible common origins of the incidence evolution.

**Results:**

The results show how neighbor municipalities tend to share a similar behavior of the disease, revealing the strong spatiotemporal relationship of the COVID-19 spreading beyond the administrative borders of each municipality. Additionally, we demonstrate how local socio-economic and demographic characteristics evolved as determinants of COVID-19 transmission, during the 1st wave school density per municipality was more relevant, where during 2nd wave jobs in the secondary sector and the deprivation score were more relevant.

**Conclusions:**

The results show that SOM can be an effective tool to analysing the spatiotemporal behavior of COVID-19 and synthetize the history of the disease in mainland Portugal during the period in analysis. While SOM have been applied to diverse scientific fields, the application of SOM to study the spatiotemporal evolution of COVID-19 is still limited. This work illustrates how SOM can be used to describe the spatiotemporal behavior of epidemic events. While the example shown herein uses 14-days cumulative incidence curves, the same analysis can be performed using other relevant data such as mortality data, vaccination rates or even infection rates of other disease of infectious nature.

## Introduction

In December of 2019, multiple cases of a highly transmittable virus, the SARS-CoV-2 virus, were identified in China’s Wuhan city, Hubei province [1]. The World Health Organization (WHO) named the disease itself as the Coronavirus Disease-2019 (COVID-19) [2]. The initial measures and strategies to combat and mitigate the propagation of the virus in China were ineffective, resulting in propagation of the virus worldwide. What was originally a local epidemic event, rapidly escalated into a global pandemic phenomenon [3]. This pandemic had serious implications in the stress of the national health systems and in terms of fatalities, which resulted directly from the virus propagation [4].

To fight and delay the propagation of the virus, and before the generalization of the vaccination, lockdowns were one of the strategies adopted by governments worldwide. The reduced economic activity during lockdown periods exacerbated existing economic and social inequalities in countries around the globe [5–7]. Portugal was not exception and in the first year of pandemic several local (i.e., per municipality or group of municipalities) and national periods of lockdown were implemented aiming to deaccelerate the growth of the COVID-19 incidence curves. Besides, these mitigation actions also comprised those aiming to reduce social gatherings, the concentration of people in closed spaces and restrict people’s mobility to their main residency area (or municipality) [8]. However, the impact of these measures in effectively preventing the virus transmission varied depending on the socio-economic and demographic characteristics of the region where they were applied [9]. Therefore, the dynamics of the virus depends simultaneously on space and time domains and consequently its modelling should be jointly performed.

Several numerical modelling tools were applied to this end. Initially, contagion risk models (e.g., SIR models [10, 11]) provided a relevant source of information for public health authorities and governments and for the strategies developed to minimize the impact of the pandemic on health systems. However, these models are difficult to calibrate locally with field data at the small-scale (e.g., per municipality or parish) as the disease spreading depends simultaneously on the individual and social behaviors [12, 13]. Along with these models, geo-spatial mapping tools were also developed and made available to the community. This set of tools included information dashboards at local and national levels, infection risk maps produce with geostatistical tools (e.g., [14]), spatiotemporal modeling and forecasting with machine learning methods based on neural networks and deep learning (e.g., [15–17]) and spatial analysis tools based on spatial correlation indices [18].

Since the outbreak of the disease large amounts of data regarding the evolution of COVID-19 were produced. These data have the potential to provide insights into the dynamics of the spatiotemporal evolution of the pandemic allowing to devise better mitigation strategies for new pandemic or epidemic events. Under this scope, we leverage machine learning methods (i.e., self-organizing maps (SOM)) to explore, analyze and classify local 14-days cumulative incidence curves of COVID-19 for each the 278 municipalities in mainland Portugal along with key socio-economic and demographic characteristics of these municipalities. We use data from the first year of the pandemic in mainland Portugal between March 15th, 2020, and February 6th, 2021 (i.e., a total of 326 days).

Self-Organizing Maps are an artificial-neural network used as a dimensionality reduction technique or as an unsupervised clustering method [19]. This algorithm performs both vector quantization and vector projection and uses a neighborhood function to preserve the topological properties of the input space [20], being a powerful dimensionality reduction algorithm, while keeping the notion of neighbor, which is important for data with a spatial continuity pattern. When applied to data spatially distributed, SOM can explain complex elements associations in a spatial perspective [21]. Besides, as similar inputs in the original high-dimension space tend to be mapped together in its low-dimension output space, SOM can represent the probability distribution of inputs patterns and encode their associations and nonlinear relationships [22].

SOM have been applied in different scientific fields, but its use in health-related studies is still limited (e.g., [23, 24]). Melin et al. [25] used SOM to spatially group countries worldwide and then all the 32 states of Mexico, according to their COVID-19 incidence rates and mortality data. Similarly, Galvan et al. [26] analyzed the evolution of the disease in regions, states, and major cities of Brazil. Galvan et al. [27] used SOM to cluster together the Brazilian Sates according to their incidence rates and death numbers along with other health indicators into the model, having concluded that the states with higher ICU beds, ventilators, physicians and nurses per 100,000 inhabitants are clustered together and less affected by COVID-19. Resta [28] used SOM as an early warning system for pandemic events in Italy considering simultaneously demographic, healthcare, and political data.

Recently, the temporal behavior of COVID-19 incidence ratio has been related to the socio-economic and demographic variables. Da Costa and Costa [29], concluded that municipalities in mainland Portugal with more elderly people in nursing homes and with a higher number of immigrants were at a higher incidence risk of COVID-19. Lewis et al. [30] proposed the Area-Level Deprivation index for the Utah state (USA) and concluded that the odds of infection by COVID-19 were two times greater in high-deprivation areas and three times greater in very high-deprivation areas. Additionally, de Lusignan et al. [31] in a cross-sectional study, analyzed the risk factors influencing the infection by SARS-CoV-2 in the United Kingdom and concluded that people living in more deprived, densely populated areas and of Black ethnicity were at higher risk of contracting the disease.

We propose herein the use of SOM to spatially explore, at the municipality level, the first year of pandemic in mainland Portugal and the influence of local socio-economic and demographic variables in the spread of the disease. We use SOM due to the ability of this algorithm to model data with different temporal resolution (i.e., 14-days incidence curves and socio-demographic indicators) while preserving the spatial nature of the data (i.e., the geographical location of the municipalities). This unique characteristic makes SOM suitable to model natural phenomena with both temporal and spatial components, like the spread of a contagious disease. The results shown herein represent one of the first attempts to interpret at the municipality level, and from a geo-spatial perspective, the influence of local socio-economic and demographic characteristics in the spread of COVID-19 in mainland Portugal.

Next, we briefly describe the theoretical background of SOM, followed by a description of the data set used in this study. Then, we present the main results of the spatiotemporal modelling of COVID-19 evolution with SOM in mainland Portugal. The last section draws the main conclusions.

## Methodology

In this section we first detail the architecture of the neural network used within the SOM applied in this work. Then, we describe the SOM parameterization. The proposed methodological approach was developed using the MiniSom library [32], one of the most popular SOM libraries in Python, alongside with NumPy and Pandas for data processing, gathering, and handling. Python’s Matplotlib, Seaborn and GeoPandas libraries were used for data visualization.

### Self-organizing maps architecture

**Fig. 1:**
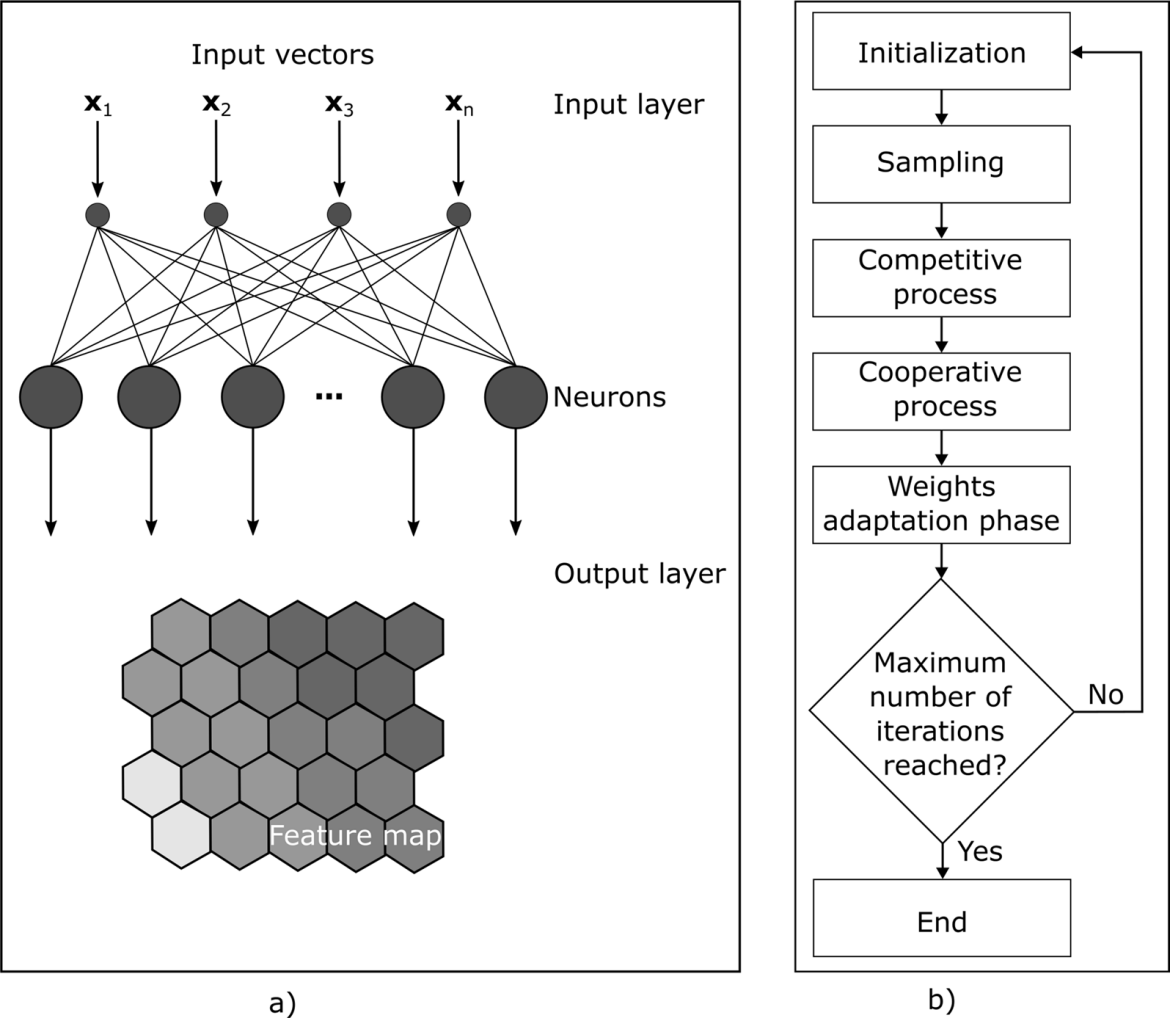
Schematic representation of: **a** the SOM architecture; **b** main steps during SOM training

Rather than from the minimization of an error between observed and predicted data (e.g., gradient descent and back-propagation), a SOM is a neural network with two layers that learns under a competitive framework. The first layer of the neural network is the input layer, which corresponds to a high-dimension noisy space (i.e., the 14-days incidence curves and the socio-demographic indicators per municipality). The second layer is the output layer and corresponds to a lower dimension than the input layer (i.e., the output feature map) (Fig. 1b).

The input space is defined has having $n$ dimensions $\mathbf{x}=[ {\mathbf{x}}_{1},{\mathbf{x}}_{2},{\mathbf{x}}_{3}\dots {\mathbf{x}}_{\mathrm{n}}]$. In the application case shown herein, $\mathrm{n}$ corresponds to the total number of municipalities considered ($\mathrm{n}=278$) and each input data vector (${\mathbf{x}}_{\mathrm{i}}$) is composed by the $t$ 14-days cumulative incidence ratio over time (i.e., time series) and $k$ socio-economic and demographic variables associated to a specific municipality of mainland Portugal. The size of each vector ${\mathbf{x}}_{\mathrm{Ii}}$ is $t+k$.

The output space is defined has having $m$ dimensions $\mathbf{w}=[{\mathbf{w}}_{1},{\mathbf{w}}_{2},{\mathbf{w}}_{3},\dots , {\mathbf{w}}_{\mathrm{m}}]$. The number of neurons in the output layer ($\mathrm{m}$) depends on the objective of the work. Each ${\mathbf{w}}_{\mathrm{j}}$ output neuron is fully connected to each $\mathrm{n}$ dimensional input data of the input layer through a connection reference weight vector defined as ${\mathbf{w}}_{\mathrm{j}}=[{\mathbf{w}}_{\mathrm{j}1},{\mathbf{w}}_{\mathrm{j}2},{\mathbf{w}}_{\mathrm{j}3}\dots {\mathbf{w}}_{\mathrm{jn}}]$, which defines each output neuron in the input space (i.e., each connection weight vector has the same number of dimensions of ${\mathbf{x}}_{\mathrm{i}}$). According to Bação et al. [33] the size of the output layer should be smaller than the size of the input layer but allowing each cluster to be represented by multiple neurons. Hence, in the application example shown herein we set the SOM output space to a 5 by 5 two-dimensional grid (i.e., $\mathrm{m}=25$ neurons). This size was achieved after testing several configuration and is a good compromise to discriminate municipalities with different behaviors, while avoiding municipalities with large differences to be clustered together.

The SOM algorithm applied in the application example shown below can be summarized in the following sequence of six steps [34] (Fig. 1b):

(i) Initialization—Initialize randomly all the weights of the connection reference weight vectors (${\mathbf{w}}_{\mathrm{j}}$). Alternative initialization methods can be used (e.g., principal component analysis);
(ii) Sampling—Select an input sample $I$ (i.e., a municipality) from the $n$ observations in the data set (i.e., from the 278 municipalities from mainland Portugal);
(iii) Competitive effects—Compute the Euclidean distance between the sampled municipality (${\mathbf{x}}_{\mathrm{i}}$) and the connection weight vector (${\mathbf{w}}_{\mathrm{j}}$) of a $\mathrm{j}$ output neuron, using all output neurons (i.e., the discriminant function)
  1 $$d\left({\mathbf{x}}_{\mathrm{i}}\right)=\sum_{\mathrm{k}=1}^{\mathrm{n}}{\left({\mathrm{x}}_{\mathrm{ik}}{-\mathrm{ w}}_{\mathrm{jk}} \right)}^{2}$$
  where ${\mathrm{w}}_{\mathrm{jk}}$ is the value in entry $\mathrm{k}$ of the connection weight vector of the $\mathrm{j}$ output neuron and ${\mathrm{x}}_{\mathrm{ik}}$ is the feature $\mathrm{k}$ value in the input sample ${\mathbf{x}\mathbf{I}}_{\mathrm{i}}$, both with $n$ dimensions. Then, the output neuron ${\mathbf{w}}_{\mathrm{j}}$ that minimizes the discriminant function (Eq. 1) (i.e., output neuron more similar to the municipality ${\mathbf{x}}_{\mathrm{i}}$) is declared the winning neuron, known as its Best Matching Unit (BMU);
(iv) Cooperative process—Compute the topological neighborhood of the BMU using a Gaussian Function (${\mathrm{h}}_{\mathrm{j},{\mathrm{x}}_{\mathrm{i}}}$) [25]
  2 $${\mathrm{h}}_{\mathrm{j},{\mathbf{x}}_{\mathrm{i}}}={\mathrm{e}}^{\frac{{-\mathrm{d}}_{\mathrm{j},\mathrm{\rm I}\left({\mathbf{x}}_{\mathrm{i}}\right)}^{2}}{2{\upsigma }^{2}}}$$

where ${\mathrm{d}}_{\mathrm{j},\mathrm{\rm I}\left({\mathbf{I}}_{\mathrm{i}}\right)}$ is the Euclidean distance between the $\mathrm{j}$ output neuron and the winning neuron, $\mathrm{\rm I}({\mathbf{x}}_{\mathrm{i}})$, for the municipality $I$, and $\upsigma$ represents the initial neighborhood radius. A $\upsigma$ of 2 indicates the neighborhood around the BMU only comprises neurons until 2 units of distance. The topological neighborhood function ${\mathrm{h}}_{\mathrm{j},{\mathbf{x}}_{\mathrm{i}}}$ assumes values between 0 and 1, having its maximum at the BMU and then monotonically decreasing until reaching the neighborhood radius $\upsigma$. It is zero for all the remaining neurons in the output space. Moreover, $\upsigma$ can be defined using an exponential decaying function

3 $$\upsigma \left(\mathrm{l}\right)={{\upsigma }_{0}}^{\frac{-\mathrm{l}}{{\uptau }_{\upsigma }}}$$

where, $\mathrm{l}$ is iteration number during the training of the SOM, ${\tau }_{\sigma }$ is a decay constant set at the beginning of the algorithm. ${\tau }_{\sigma }$ is usually set equal to the number of iterations. The purpose of this decaying function is ensuring the neighborhood radius, that initially can go up to the size of the output space, decreases with time, eventually converging to zero, which becomes important as the training process enters the convergence phase;

(xxii) Weights adaptation phase—the learning process happens through updating the weight connection vectors. At this step, both the BMU and its neighboring neurons have their weight connection vectors updated following [25]: where $\alpha$ is the learning rate, ${\mathrm{h}}_{\mathrm{I},{\mathbf{x}}_{\mathrm{i}}}$ is the topological neighborhood computed in the last step (Eq. 2). The result of applying this formula is moving the connection weight vectors of the BMU and its neighborhood closer to the municipality ${\mathbf{x}}_{\mathrm{i}}$. This is what allows SOM to perform a topological mapping where the initial topology of the input space is kept, since similar municipalities in the initial high-dimensional space end-up being mapped to SOM neurons close in the low-dimensional space [21]. Additionally, the learning rate, α, which determines for how much the connection weights are adjusted, is defined following
  5 $$\mathrm{\alpha }\left(\mathrm{l}\right){{\mathrm{\alpha }}_{0}}^{\frac{-\mathrm{l}}{{\uptau }_{\mathrm{\alpha }}}}$$
  where, $\mathrm{l}$ is iteration number during the training of the SOM, ${\tau }_{\alpha }$ is a decay constant set initially. Hence, the learning rate decreases over time until eventually converges to zero, which is essential in the convergence phase of training to ensure the training vectors fed into SOM are contributing to its output layer refinement, rather than just obliterating the learning of previous iterations [25].
  4 $$\Delta {\mathbf{w}}_{\mathrm{j}}=\mathrm{\alpha }\left(\mathrm{t}\right){\mathrm{h}}_{\mathrm{j},{\mathbf{x}}_{\mathrm{i}}}\left({\mathbf{x}}_{\mathrm{i}}- {\mathbf{w}}_{\mathrm{j}}\right)$$

(vi) Continuation–phase—Repeat iteratively all the steps from ii) to v). Updating the learning rate and neighborhood size after each iteration. The iterative procedure stops when the number of training iterations initially defined is reached.

## Evaluation of the quality of the SOM output space

After the training phase of the SOM is concluded, it is crucial to evaluate the quality of the predicted output feature map. The output feature map should describe the non-linear associations and properties of the input data set. In SOM the quality of the output feature space is assessed by the quantization and topographic errors present in its output space. The quantization error (QE) is the average Euclidean distance between each municipality, ${\mathbf{x}}_{\mathrm{i}}$, of the $n$ municipalities and their BMU’s weights vector (${\mathbf{w}}_{\mathrm{\rm I}\left({\mathbf{x}}_{\mathrm{i}}\right)}$)

6 $$\mathrm{QE}=\frac{{\sum }_{\mathrm{i}=1}^{\mathrm{n}}{\left({\mathbf{x}}_{\mathrm{i}}- { \mathbf{w}}_{\mathrm{\rm I}\left({\mathbf{x}}_{\mathrm{i}}\right)}\right)}^{2}}{\mathrm{n}}$$

Ideally, QE is as small as possible.

The Topological Error (TE) measures SOM’s ability to preserve the initial topology of the input data in its output space (i.e., similar municipalities are mapped together or to neighbor neurons). The $\mathrm{TE}$ is given by the total errors among all municipality’s mappings divided by the $n$ municipalities. Being considered an error when for municipality ${\mathbf{x}}_{\mathrm{i}}$ its BMU and the second BMU are not neighbor neurons

7 $$\mathrm{TE}=\frac{{\sum }_{\mathrm{i}=1}^{\mathrm{n}}\mathrm{f}\left({\mathbf{x}}_{\mathrm{i}}\right)}{\mathrm{n}}$$

where,

8 $$f(x_{i} ) = \left\{ {\begin{array}{*{20}l} 0 \hfill & {same\;BMU\;and\;2{{nd}} \;BMU} \hfill \\ 1 \hfill & {otherwise} \hfill \\ \end{array} } \right.$$

Thus, TE is equal to 1 when none of the initial topology is preserved. Therefore, we aim at the smallest TE possible.

## SOM parameterization

**Fig. 2:**
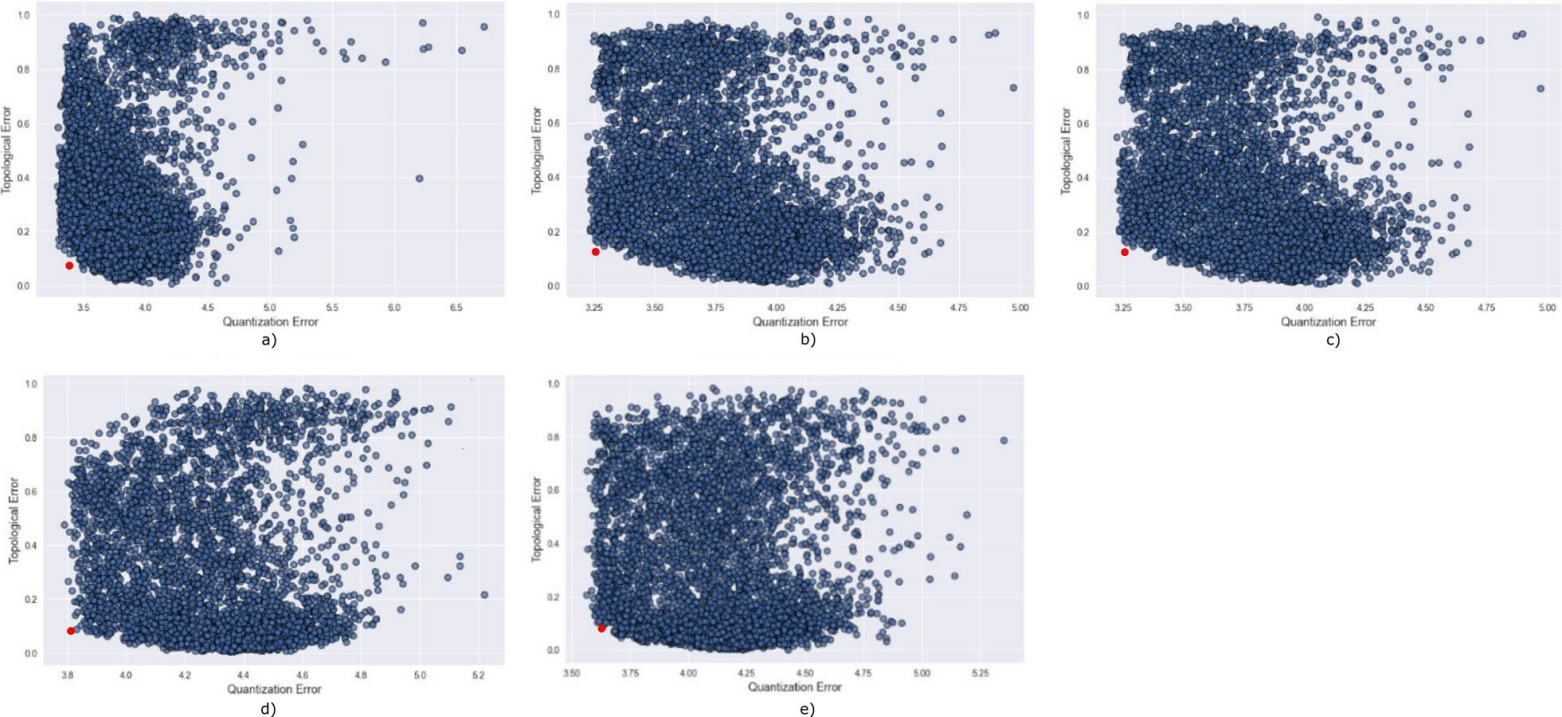
QE and TE for all the 16,400 SOM models evaluated during the SOM parameterization (blue filled circles) for the five periods considered during the first year of pandemic: **a** 1st emergency state; **b** summer season; **c** sept-oct of 2020; **d** 2nd wave of COVID-19; **e** Holyday season. The model with the smallest QE and TE is highlighted in red

The choice of parameters prior to the training phase of the SOM is instrumental for the quality of the results obtained. These parameters comprise the learning rate (${\mathrm{\alpha }}_{0}$), neighbor radius (${\upsigma }_{0}$) and number of iterations (${\mathrm{l}}_{\mathrm{tot}}$), along with the sampling strategy and the weights initialization. In the application example shown below, these parameters were set after evaluating a total of 16,400 SOM models (Fig. 2). Each SOM model was obtained by changing one parameter at the time following the ranges and increments summarized in Table 1. We selected the SOM models with the smallest QE and TE, represented by the red filled circle in Fig. 2.

**Table 1:** Range and increment of the SOM parameters tested to select the best SOM model

Parameter	Range
${\mathrm{\alpha }}_{0}$	Range: 0.2–2 Increment: 0.2
${\upsigma }_{0}$	Range: 0.2–2 Increment: 0.2
${\mathrm{l}}_{\mathrm{tot}}$	Range: 1000–5000 Increment: 100

## Visualization of SOM’s output space

The standard way to visualize the SOM output space is using a unified distance matrix (U-Matrix) [35], in which a color gradient-dependent on the Euclidean distance between neurons is applied to allow the identification of clusters. Light areas indicate high similarity between neighbor neurons, and therefore a possible cluster, while darker represent clusters with different behavior from the main trend [36]. In the U-matrix plot, and for each output neuron, we add a point every time a municipality is mapped into that neuron. This approach allows the U-Matrix to display the activation frequencies of neurons.

Additionally, we used components planes to evaluate the weight vectors values per neuron for specific input features. In the application example shown herein, we use component planes to assess the importance of the local socio-economic and demographic variables in spatiotemporal evolution of COVID-19 in mainland Portugal.

**Fig. 3:**
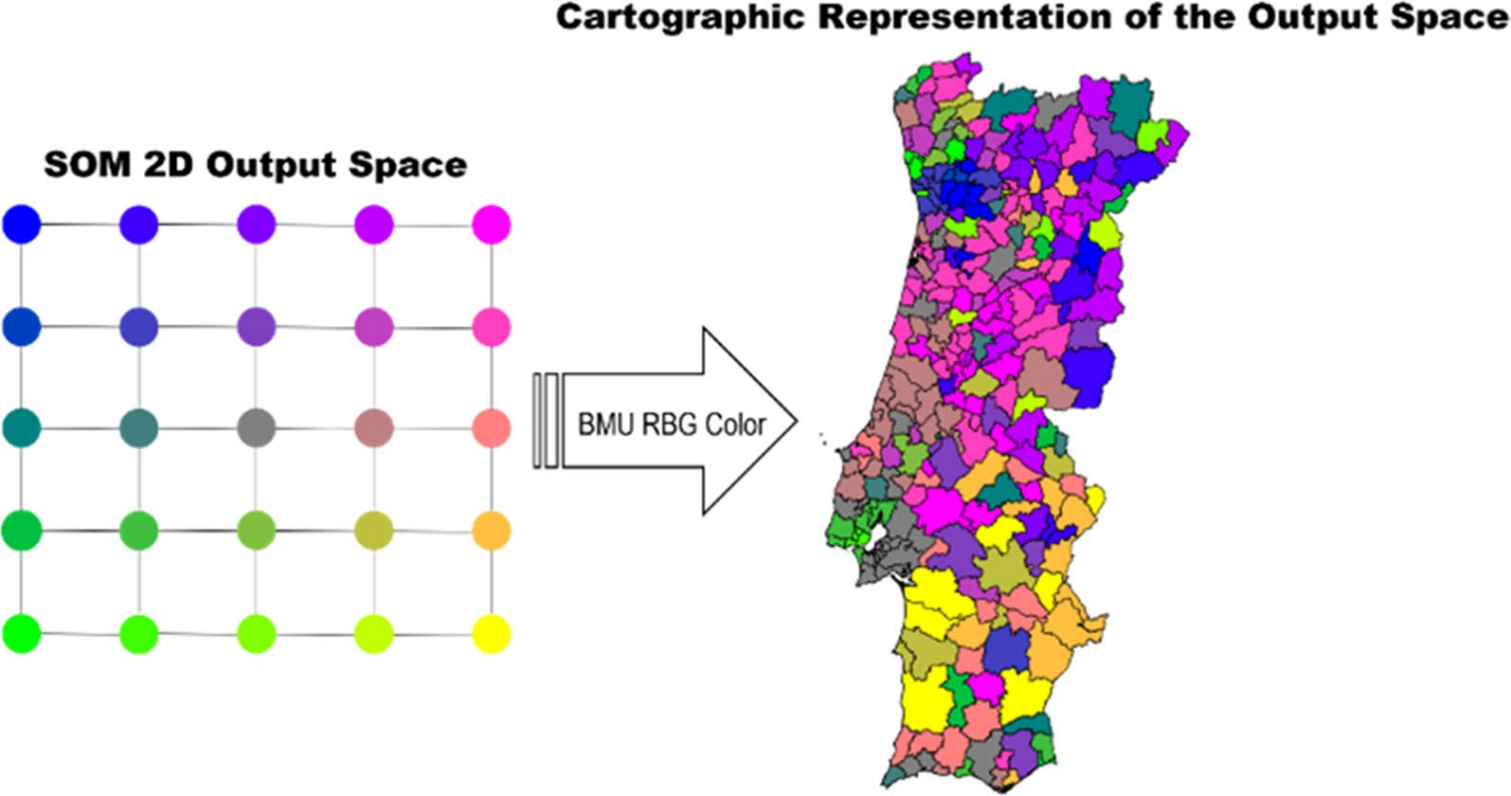
Schematic representation of the geographical representation of the SOM output feature space using projected coordinate system for each municipality

**Fig. 4:**
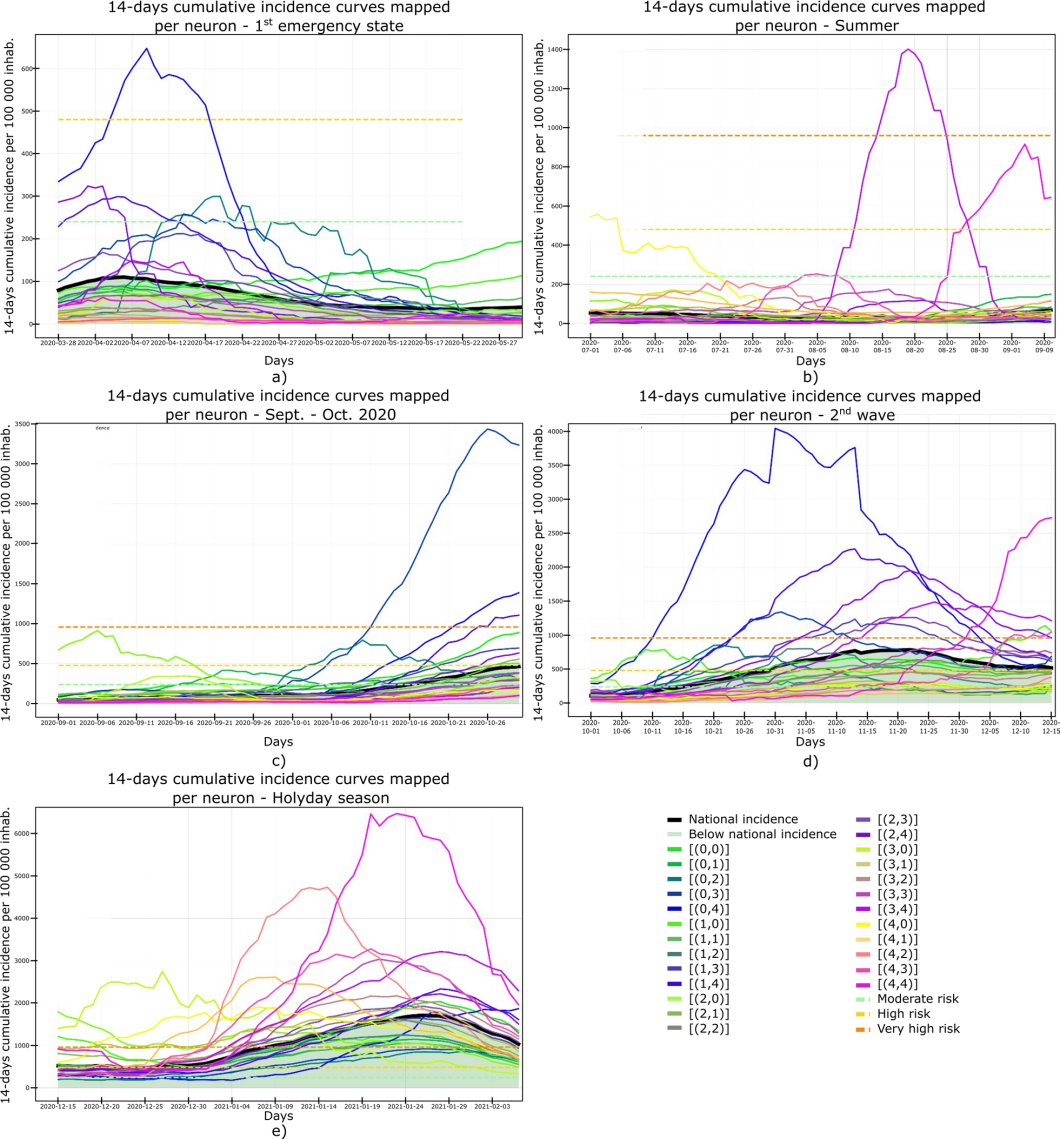
14-days cumulative incidence curves for all the municipalities considering: **a** 1st emergency state; **b** summer season; **c** sept-oct of 2020; **d** 2nd wave of COVID-19; **e** Holyday season. Each color represents a different municipality as shown in Figs. 5 and 6. Black thick curve represents the national 14-days cumulative incidence curve

As municipalities have intrinsic geo-spatial properties, we also show the output feature map projected in a cartographic view (i.e., using a projected coordinate system for Portugal). We follow a similar approach to Gorricha and Lobo [37] and assign a unique combination of Red, Green and Blue (RGB) colors to each neuron while preserving the SOM topological features by making adjacent neurons share similar colors (Fig. 4).

For a generic output space of $N\cdot M$ neurons each neuron has ($x,y$) coordinates in the range:

9 $$\genfrac{}{}{0pt}{}{x=\mathrm{0,1},\dots N-1}{y=\mathrm{0,1}\dots M-1}$$

Each neuron with coordinates ($x,y$) will be assigned a RBG color given by:

10 $$R=\frac{x}{N-1}$$

11 $$G=1- \frac{y}{M-1}$$

12 $$B=\frac{y}{M-1}$$

## Application example

### Data set description

The main objective of this work is to study the spatiotemporal evolution of COVID-19 in mainland Portugal using SOM. To this end, we use data from the Portuguese Epidemiological Surveillance System (SINAVE). SINAVE is a mandatory national web-based surveillance system that registers all SARS-CoV-2 cases, in Portugal mainland. A SARS-CoV-2 case corresponds to a laboratory-confirmed SARS-CoV-2 infection reported in SINAVE. According to the case definition, both Polymerase Chain Reaction (PCR) and Rapid Antigen Test (RAT) can be used for diagnostic purposes. For the geographical allocation of SARS-CoV-2 cases, we used the municipally of the confirmed test or, when missing, the address of residence of the case registered in the national patient’s database. The study period comprises the daily confirmed number of SARS-CoV-2 cases of reported between March 28^th^, 2020, and February 6th, 2021, for each municipality located in mainland Portugal.

From these data we computed the 14-days cumulative incidence rate per 100,000 inhabitants. The dataframe with the cumulative incidence rate data used as part of the input of the SOM has the structure illustrated in Table 2, where each column corresponds to a specific day $d$ with $d=\mathrm{1,2}, \dots , 326$ and each row indicates a municipality $n$ with $n=1, 2,\dots , 278$. Each row in the final dataframe can be seen as a time series data.

**Table 2:** Fourteen-days cumulative incidence dataframe used as input for the SOM

	$d = 1$	$d = j$	$d=326$
$n = 1$	14-days cumulative incidence rate per 100,000 $(n = 1, d = 1)$	14-days cumulative incidence rate per 100,000 $(n = i, d = j)$	14-days cumulative incidence rate per 100,000 $(n = 1, d = 326)$
$n = i$	14-days cumulative incidence rate per 100,000 $(n = i, d =1)$	14-days cumulative incidence rate per 100,000 $(n = i, d = j)$	14-days cumulative incidence rate per 100,000 $(n = i, d = 326)$
$n =278$	14-days cumulative incidence rate per 100,000 $(n = 278, d =1)$	14-days cumulative incidence rate per 100,000 $(n = i, d = j)$	14-days cumulative incidence rate per 100,000 $(n = 278, d = 326)$

Additionally, for each of the 278 municipalities, nine additional features of socio-economic and demographic nature were gathered from Statistics Portugal (INE, https://www.ine.pt/), PORDATA (https://www.pordata.pt), both public domain data repositories and the deprivation score developed within the scope of the European Deprivation Index project and based on Portugal’s census data from 2011 [38] (Table 3). The deprivation score summarizes the poverty level for each of the municipalities considering multiple different socio-economic and demographic indicators. We consider this set of features good descriptors of the socio-economic dynamics of each municipality, covering the population density and type, the type of employment and the poverty level of the population. Besides, these variables have been correlated with the COVID-19 evolution elsewhere [30].

**Table 3:** Socio-economic and demographic features used in SOM

Feature	Year	Description
Population density	2018	Inhabitants by km^2^
Deprivation score	2011	Measures poverty level
Youth population	2018	% of Inhabitants 0–19 years
Elderly population	2018	% of Inhabitants 65 + years
Jobs in the primary sector	2014	% of Working population in primary sector
Jobs in the secondary sector	2014	% of Working population in secondary sector
jobs in the tertiary sector	2014	% of Working population in tertiary sector
People in state benefits	2018	Proportion of guaranteed minimum income beneficiaries
Number of schools	2018	Number of schools/Km^2^

The 14-days COVID-19 cumulative incidence time series were submitted to an exploratory data analysis (EDA). The EDA aims to understand the temporal characteristics of the time series (i.e., how it varies throughout the first year of the pandemic) and recognize temporal patterns. After EDA, the one year long 14-days incidence curves were split in five distinct periods (Table 4). This division corresponds to different behavior of the disease within the period considered and allows a better understanding of the SOM output.

**Table 4:** The five different periods used to split the original cumulative incidence time series

Period	Name	Main events
March 28th to May 30th, 2020	1st Emergency State	• Initial mandatory national lockdown and closing of most economic sectors • Eastern Restrictions applied from April 9th to 13th, 2020
July 7th to September 10th, 2020	Summer 2020	• August 3rd the first day without COVID-19 related deaths • Some restrictions were lifted as COVID-19 cases remain low
September 1st to October 30th, 2020	September–October of 2020	• Schools reopen on September 14th, 2022 • Mandatory use of mask indoor and outdoor
November 1st to December 15th, 2020	2nd Wave of COVID-19	• Emergency State declared again on November 9th, 2022 • New daily record of COVID-19 infections on November 19th, 2022
December 15th, 2020 to February 6th, 2021	Holiday Season	• Some mobility restrictions lifted to enable Holidays celebrations • National lockdown applied again on January 18th, 2021

As the input data set include incidence cumulative data along with nine socio-economic and demographic variables, the data were rescaled to have zero mean and standard deviation of one, to avoid biases in the SOM application. In this way, we ensure the SOM weights equally each input features.

## Results

The results shown in this section were obtained with the SOM model that resulted in the smallest QE and TE from all the 16,400 models evaluated (Fig. 2). The 14-days cumulative incidence curves for all the municipalities in mainland Portugal and the five periods considered are shown in Fig. 4. Besides, this figure includes the national 14-days cumulative incidence curve, which allows comparing the behavior of each municipality against the national behavior of the disease. The five periods exhibit patterns with distinct behavior. The 14-days cumulative incidence curves tend to increase during the period considered (i.e., the 326 days), with a peak at the beginning of the pandemic and a slow decreasing for the first period (Fig. 4a), a relative flat and homogeneous behavior for the second (Fig. 4b) and third periods (Fig. 4c) with exception to a few municipalities located in coordinates (3,4) and (4,4) of the output feature map for the second period (Fig. 4b) and located in coordinate (0,4) for the third period (Fig. 4c) and a rapid growth of the incidence curves for the fourth and fifth periods (Fig. 4d and e). With the incidence curves we can understand the temporal dynamics of the disease, but its spatial evolution is not easily interpreted as it is not straightforward to reduce the dimension of each time series into a single value that can be used to visualize the data spatially.

**Fig. 5:**
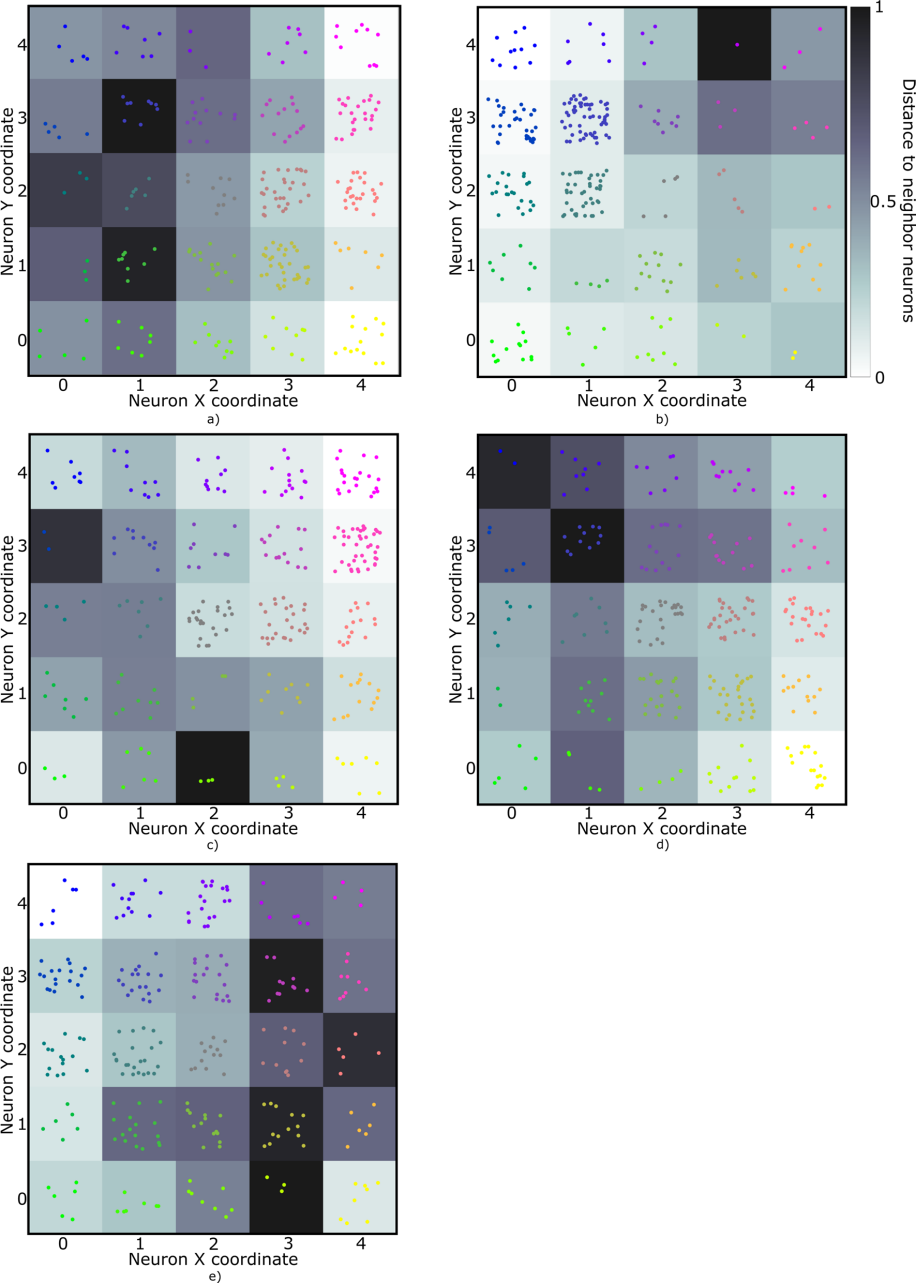
U-Matrices obtained with the SOM applied to data corresponding to the: **a** 1st emergency state; **b** summer season; **c** sept-oct of 2020; **d** 2nd wave of COVID-19; **e** Holyday season. Each color represents a different municipality as shown in Figs. 4 and 6

**Fig. 6:**
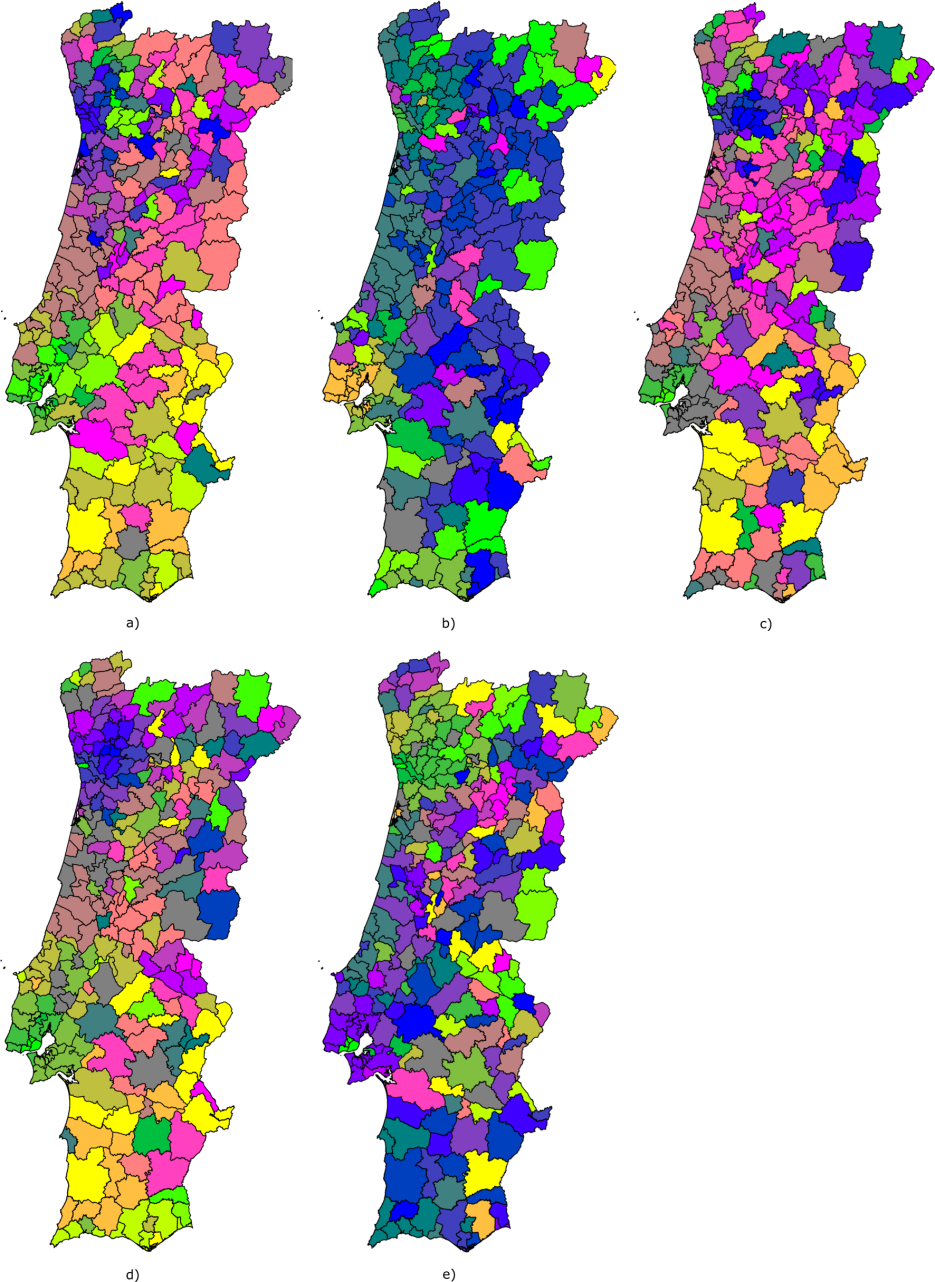
Geographical projection of the cluster obtained from the SOM models: **a** 1st emergency state; **b** summer season; **c** sept-oct of 2020; **d** 2nd wave of COVID-19; **e** Holyday season. Each color represents a different municipality

**Fig. 7:**
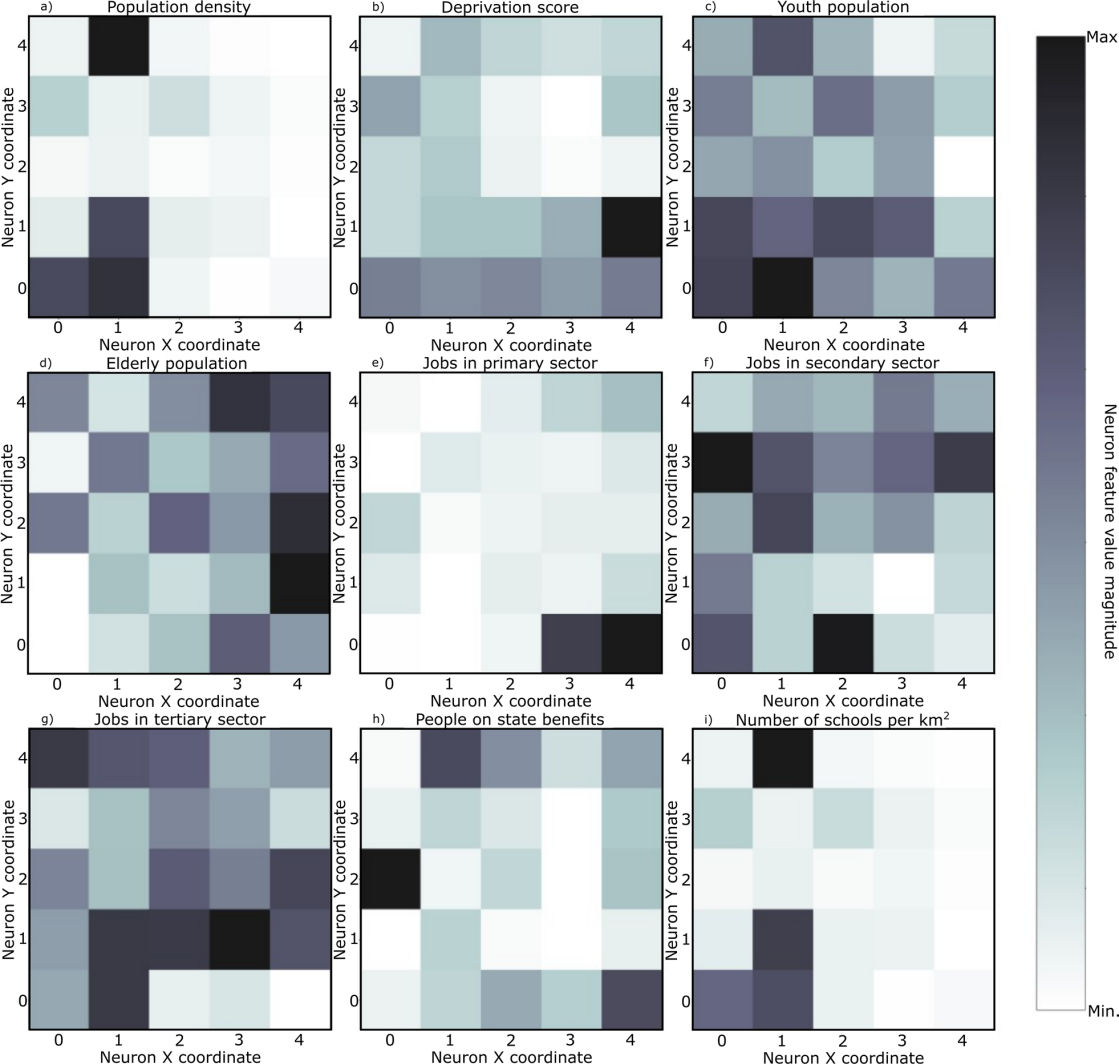
Components plane for the period corresponding to the 1st emergency state

**Fig. 8:**
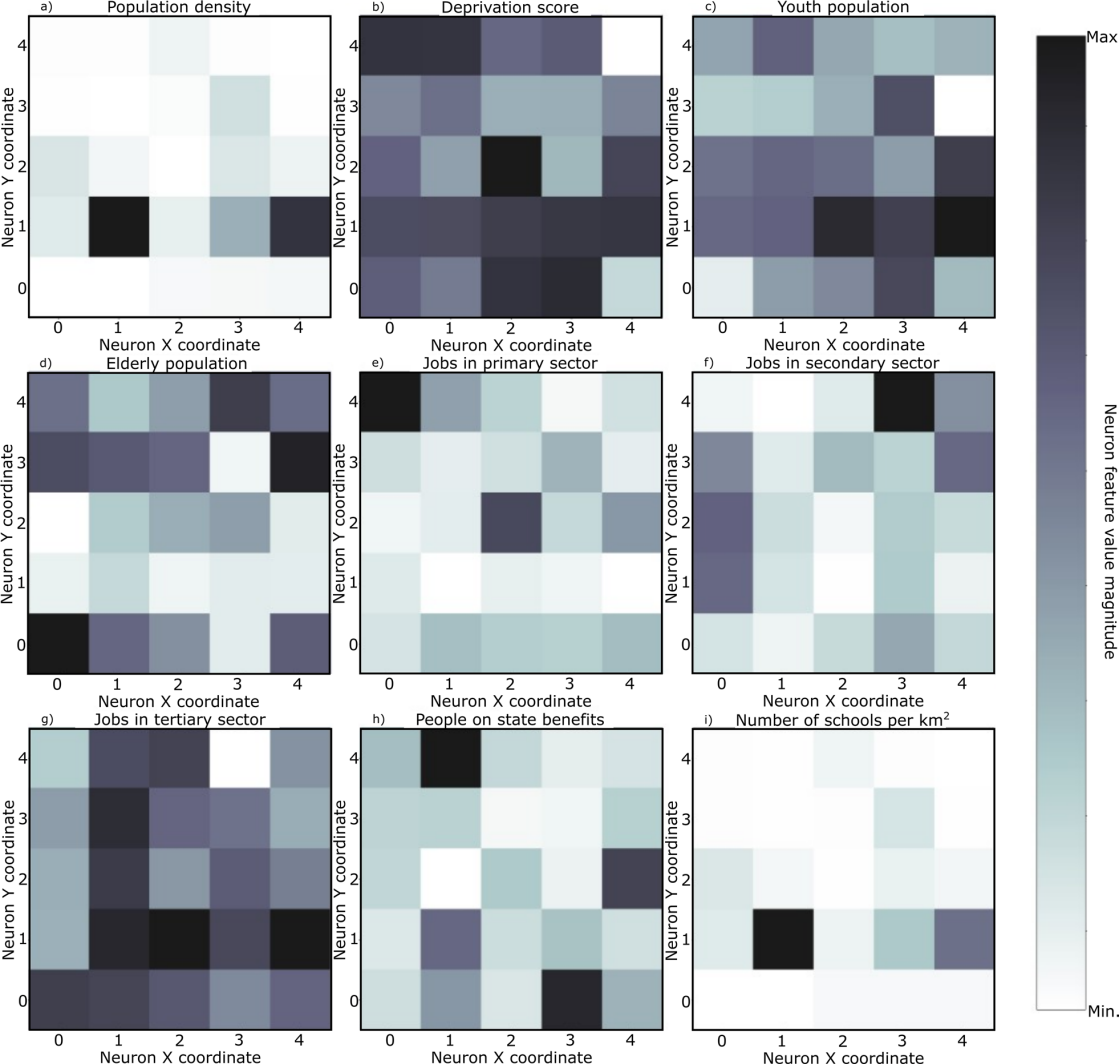
Components plane for the period corresponding to the summer season

**Fig. 9:**
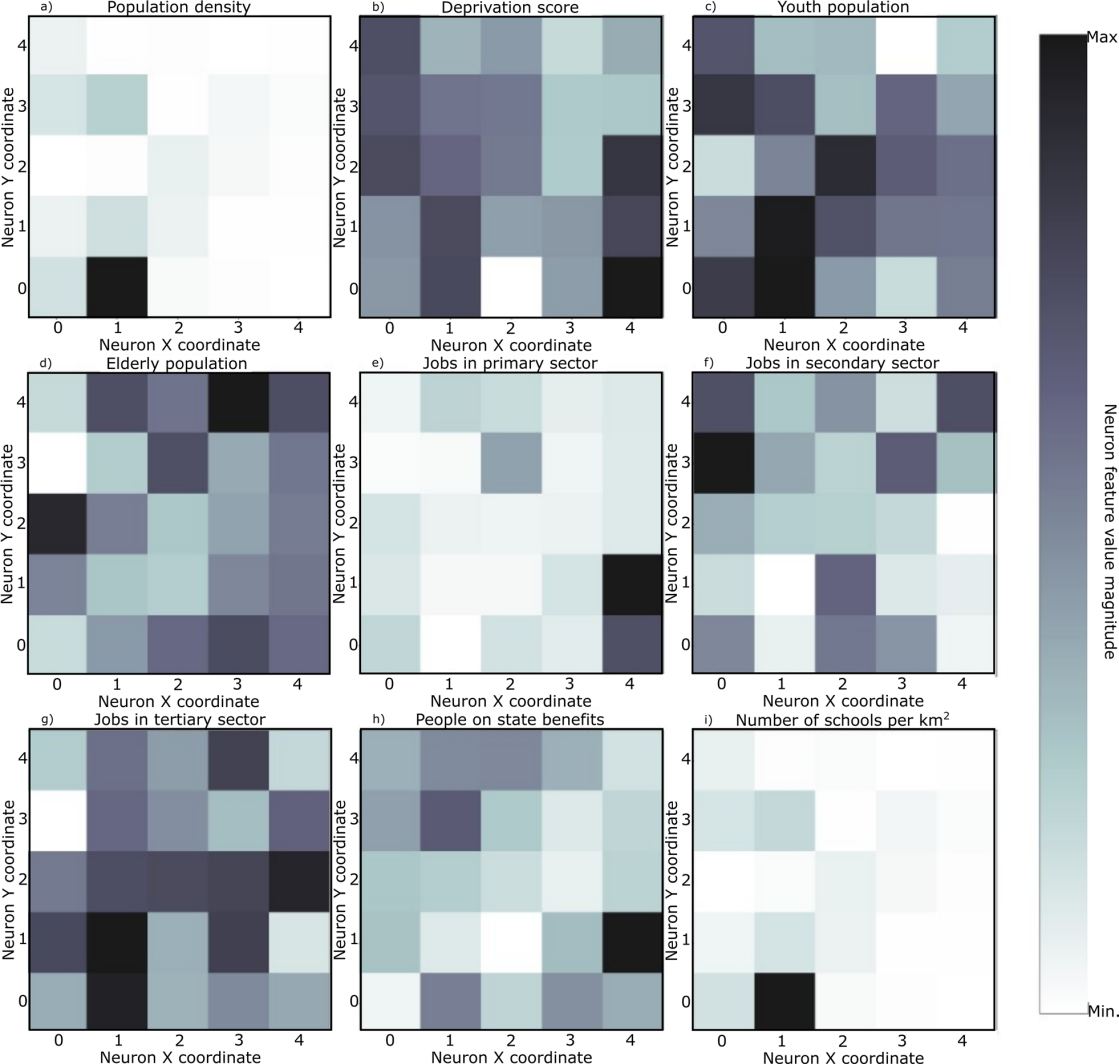
Component planes for the period corresponding to sept–oct of 2020

**Fig. 10:**
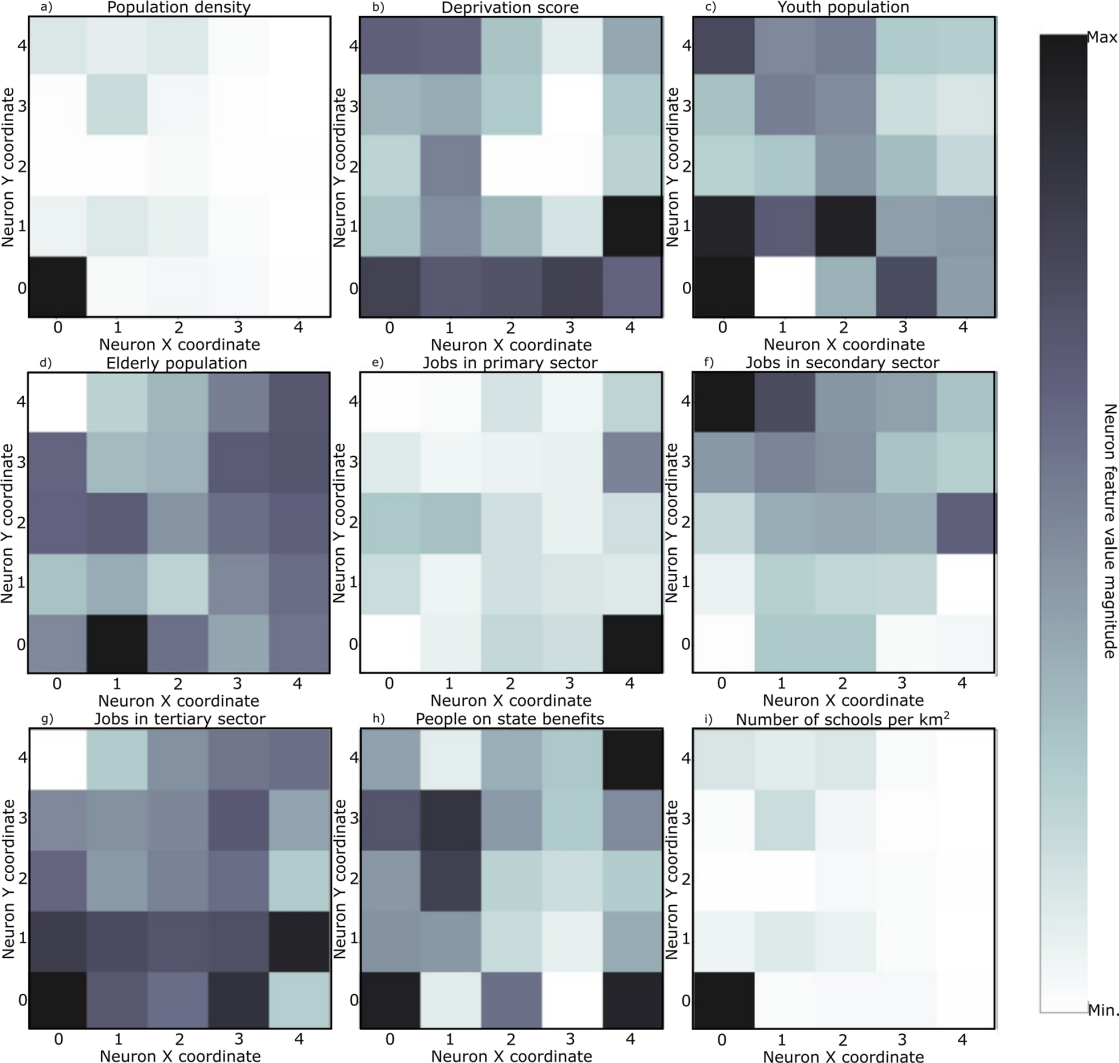
Component planes for the period corresponding to the 2nd wave of COVID-19

**Fig. 11:**
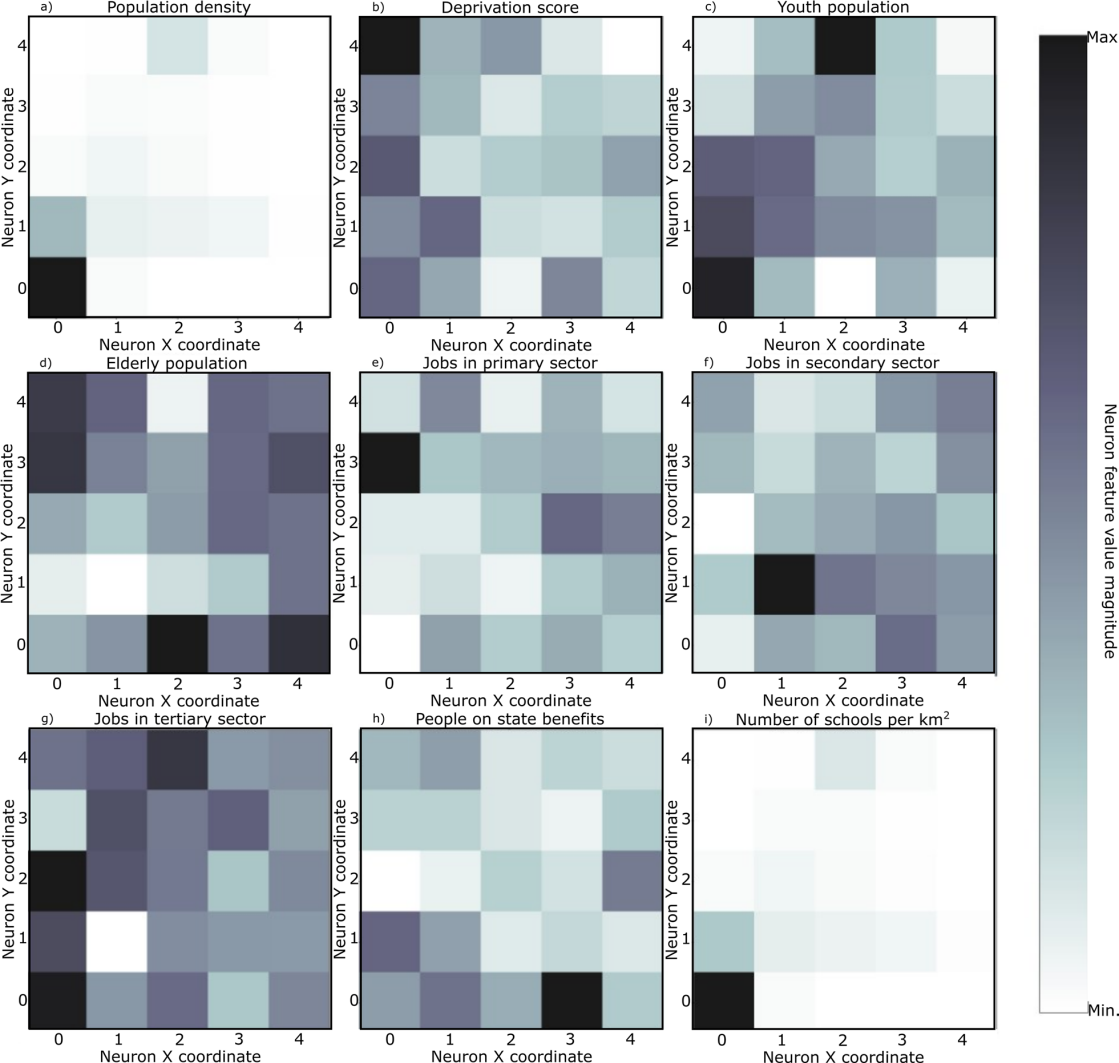
Component planes for the period corresponding to the Holyday season

SOM models were trained for each set of curves shown in Fig. 4 plus the socio-economic and demographic indicators (Table 3). The resulting U-matrix per period are shown in Fig. 5 along with the municipalities that hit each neuron using the color code described in Fig. 3. The geographical projection of these results is shown in Fig. 6 and the corresponding component planes in Figures. 7, 8, 9, 10, 11.

### 1st period: March 28th to May 30th, 2020

This period corresponds to the first emergency state and mandatory national lockdown. Most economic sectors were closed and a reinforcement of restrictive measures were applied during Easter (April 9th to 13th, 2020). For this period (Figs. 5a and 6a) there are two main clusters of municipalities that standout from the rest of the domain considered. These clusters are located in coordinates (1,1) and (1,3) in the U-Matrix (Fig. 5a) and correspond to the two main metropolitan regions in mainland Portugal (i.e., Lisbon and Porto). From the component plane projection (Fig. 7), the most relevant socio-economic and demographic variables are the number of schools, youth population and population density. These features are indeed a good summary of the social-economic and demographic characteristics of the municipalities belonging to the Lisbon’s and Porto’s metropolitan areas. The municipalities plotted along the neurons located in X-coordinate 4 (Fig. 5a) are mainly located in the Eastern region of the country (Fig. 6a), which correspond largely to an elderly population (Fig. 7). Early in the pandemic introduction of SARS-CoV-2 come mainly from highly connected areas as metropolitan areas. The results summarized by the SOM output are consistent with previous literature [39, 40]. Association with elderly population can be explained by large outbreaks in long-term care facilities [41], which are preferably located in sparse populated municipalities. For this reason, these local outbreaks highly influence the overall incidence of the municipality.

### 2nd period: July 7th to September 10th, 2020

The second timeframe considered corresponds to the months of summer and a period of relatively low incidence and a sudden burst associated with a nursing house in a small municipality (Figs. 5b, 6b and 8). This municipality is classified per se as a single cluster in the U-Matrix (Fig. 5b, located in coordinate (3,4)). Other municipalities that exhibit similar incidence curves at the end of the period considered (i.e., with a sudden bursts of confirmed cases) are mapped in the same region of the feature space. From a spatial perspective (Fig. 6b) these municipalities are dispersed along the country as these events were dependent on the local characteristics and do not have a continuous spatial continuity pattern. In fact, most of the municipalities are plotted with similar colors as they are mapped in the same region of the feature space. Also of interest is the mapping of the municipalities plotted with yellowish colors in Fig. 6b that are located within the Lisbon metropolitan area and describe the behavior of the disease after the main wave. The incidence in this region took longer to decrease comparatively to the rest of the country. As the high incidence values are associated with specific cases these do not clearly correlate with any socio-economic or demographic variable. The neurons that map the municipality with the largest outburst (located in coordinate (3,4) in Fig. 5b) in terms of socio-economic and demographic variables are related to elderly population and jobs in secondary sector (Fig. 8), which agrees with the characteristics of this region. The Northern region of Portugal is characterized by a large influence of the manufacturing industry. Moreover, industry workers never stopped working, even during lockdowns, and their jobs are mostly incompatible with remote work. Therefore, making them more vulnerable relative to the general population.

### 3rd period: September 1st to October 30th, 2020

For the third period considered, the U-Matrix (Fig. 5c) shows the presence of a big cluster, identified by the large region plotted in light colors. Besides, the activation frequencies of the neurons belonging to that cluster are much higher than in the remaining neurons of the output space. The cartographic projection of the output space (Fig. 6c) shows that this cluster covers a wide region of the territory, from the southern part up to the northern part of the country (i.e., neurons plotted in pink, brown and purple colors). These pattern reveals a spatially homogeneous behavior of the disease with low incidence 14-days incidence ratios (Fig. 4c). However, and additional class of municipalities can be identified by the dark colors of the output space (Fig. 5c). These municipalities are mainly plotted in the neurons located in coordinates (0,3), (0,2), (1,2), and (2,3) and correspond mainly to both Porto and Lisbon metropolitan areas. These areas had a distinct behavior of the diseases comparatively to the other municipalities of the country. The incidence curves for this last cluster of municipalities show larger values for longer periods of time (Fig. 4c).

The component planes for this period (Fig. 9) show that the municipalities with higher incidence values (plotted in darker color in the U-Matrix) are mainly associated with jobs in the secondary and tertiary sectors and a younger population. The increase of the incidence rates in these regions during this period might be related to the return to work of the active population of the country. However, Fig. 9 also shows the influence of the number of schools associated with some of these municipalities, suggesting a relationship between the opening of schools due to the beginning of the academic year and the disease spatiotemporal evolution.

### 4th period: November 1st to December 15th, 2020

During the fourth period there is a group of municipalities that have a clear distinctive behavior when looking at the 14-days incidence curves (Fig. 4d). This behavior is mapped in the U-Matrix (Fig. 5d) with two neurons showing a different behavior from the rest (coordinates (0,4) and (1,3)). When projected in their true geographical location, the municipalities that activated these neurons are mainly concentrated in the Porto metropolitan region (Fig. 6d). The remaining municipalities exhibit a smooth and spatially continuous values (i.e., municipalities plotted in lighter color in the U-Matrix plot).

The socio-economic and demographic factors that seem to influence the high-incidence municipalities (Fig. 10) are associated with a younger population, jobs in the secondary sector and the deprivation score. During this period Portugal adopted a tier lockdown system, with high geographical heterogeneity of non-pharmacological interventions [Resolução do Conselho de Ministros n.º 92-A/2020, (2020)].

### 5th period: December 15th, 2020 to February 6th, 2021

The last period considered comprises the highest incidence values observed for the entire time series (Fig. 4e). The output feature space (Fig. 5e) is composed of e darker areas and there it is difficult to identify clusters. This behavior indicates that for this period, the 14-days cumulative incidence is less spatially homogeneous (i.e., more spatially variable). Additionally, the activation frequencies of the neurons in the output space are also distributed homogeneously. The geographical projection of these data (Fig. 6e) shows that the Porto and Lisbon metropolitan areas are plotted in the same region of the output space indicating a similar behavior of the disease for these municipalities, which is considerably different from the remaining municipalities of the country.

The component planes (Fig. 11) show an apparent large correlation with people on state benefits for Lisbon metropolitan area, while the northern municipalities do not have a clear correlation with any of the factors considered. These municipalities are in general characterized by jobs in the primary sector with young and elder population.

## Final remarks

We used SOM to summarize the spatiotemporal dynamics of the first year of pandemics by COVID-19 in mainland Portugal. To help interpreting the results, the long 14-days incidence time series were split in 5 main periods that represent distinct evolution moments of the disease and different administrative measures to contain the evolution of the disease. The SOM were used to cluster municipalities with similar behavior simultaneously in their 14-days incidence curves and their socio-economic and demographic characteristics. We project the high-dimension data (i.e., the input data) into a two-dimensional domain composed of 25 neurons, which allows the identification of clusters and their back-projection in the true geographical coordinates.

Despite the complex behavior of the disease, which depends simultaneously on individual and group behavior, the application of SOM allowed to summarize important characteristics in the first year of pandemic in mainland Portugal. We demonstrate the uniqueness of highly populated metropolitan areas (i.e., Lisbon and Porto) in the disease transmission dynamics. The municipalities belonging to these regions often exhibited a distinct behavior from the remaining municipalities. These two regions are the most populated areas in the country with complex socio-economic interactions and with the largest density of younger population. Also, the clusters that are formed for the five periods show the heterogeneity across space and time of the disease evolution. SOM also showed its potential to isolate municipalities that suffered from outbreaks in long-term care facilities that happened mainly during the first wave of the pandemic (i.e., the first period considered).

The analysis of the component planes (Figs. 7, 8, 9, 10, 11) allows to identify the socio-economic and demographic variables that most impact the clustering obtained with SOM. The results obtained allow interpreting that the municipalities with high incidence values during the first year of pandemic are those with a large number of secondary/industry workers. These results suggest that socio-economic fabric of a given municipality does impact the incidence of the disease.

While the application example shown herein uses 14-days cumulative incidence curves, the same type of analysis can be performed using other relevant sources of information such as mortality data, vaccination rates or even infection rates of other disease of infectious nature.

Finally, our results cannot claim causality between the explanatory variables and the COVID-19 dynamics. However, SOM methods can be used in the future for hypothesis generation or to inform policy if no better evidence is available.

## Data Availability

The data that support this study are available from the authors upon reasonable request and with permission of DGS.
